# RNA-Seq analysis of ileocecal valve and peripheral blood from Holstein cattle infected with *Mycobacterium avium* subsp. *paratuberculosis* revealed dysregulation of the CXCL8/IL8 signaling pathway

**DOI:** 10.1038/s41598-019-51328-0

**Published:** 2019-10-16

**Authors:** Marta Alonso-Hearn, Maria Canive, Cristina Blanco-Vazquez, Rosana Torremocha, Ana Balseiro, Javier Amado, Endika Varela-Martinez, Ricardo Ramos, Begoña M. Jugo, Rosa Casais

**Affiliations:** 1 NEIKER-Instituto Vasco de Investigación y Desarrollo Agrario, Animal Health Department, Derio, Bizkaia Spain; 2 SERIDA, Servicio Regional de Investigación y Desarrollo Agroalimentario, Centro de Biotecnología Animal, Deva, Asturias Spain; 3 Scientific Park of Madrid, Genomic Unit, Campus de Cantoblanco, Madrid, Spain; 4 LSAPA, Animal Health Laboratory of the Principality of Asturias, Department of Microbiology, Gijón, Asturias Spain; 5 University of the Basque Country (UPV-EHU), Department of Genetics, Physical Anthropology and Animal Physiology, Faculty of Science and Technology, Bilbao, Spain

**Keywords:** Gene expression profiling, Bacterial host response, Bacterial pathogenesis, Gene expression analysis

## Abstract

Paratuberculosis is chronic granulomatous enteritis of ruminants caused by *Mycobacterium avium* subsp. *paratuberculosis* (MAP). Whole RNA-sequencing (RNA-Seq) is a promising source of novel biomarkers for early MAP infection and disease progression in cattle. Since the blood transcriptome is widely used as a source of biomarkers, we analyzed whether it recapitulates, at least in part, the transcriptome of the ileocecal valve (ICV), the primary site of MAP colonization. Total RNA was prepared from peripheral blood (PB) and ICV samples, and RNA-Seq was used to compare gene expression between animals with focal or diffuse histopathological lesions in gut tissues versus control animals with no detectable signs of infection. Our results demonstrated both shared, and PB and ICV-specific gene expression in response to a natural MAP infection. As expected, the number of differentially expressed (DE) genes was larger in the ICV than in the PB samples. Among the DE genes in the PB and ICV samples, there were some common genes irrespective of the type of lesion including the *C-X-C motif chemokine ligand 8* (*CXCL8/IL8*), *apolipoprotein L* (*APOLD1*), and the *interferon inducible protein 27* (*IFI27*). The biological processes (BP) enriched in the PB gene expression profiles from the cows with diffuse lesions included the killing of cells of other organism, defense response, immune response and the regulation of neutrophil chemotaxis. Two of these BP, the defense and immune response, were also enriched in the ICV from the cows with diffuse lesions. Metabolic analysis of the DE genes revealed that the N-glycan biosynthesis, bile secretion, one-carbon pool by folate and purine metabolism were significantly enriched in the ICV from the cows with focal lesions. In the ICV from cows with diffuse lesions; the valine, leucine and isoleucine degradation route, purine metabolism, vitamin digestion and absorption and the cholesterol routes were enriched. Some of the identified DE genes, BP and metabolic pathways will be studied further to develop novel diagnostic tools, vaccines and immunotherapeutics.

## Introduction

Johne’s disease or paratuberculosis (PTB) is a chronic granulomatous enteritis of ruminant animals caused by *Mycobacterium avium* subsp. *paratuberculosis* (MAP). Transmission of MAP primarily occurs by the fecal-oral route through the ingestion of MAP contaminated feces, colostrum, or milk. Infection usually occurs within the first months of live of the animal but remains subclinical for an average of 2–5 years. After being ingested, MAP crosses the intestinal mucosa where it is phagocytosed by sub-epithelial macrophages establishing a chronic infection^1^. MAP is able to survive and proliferate within phagosomes by inhibiting apoptosis and phagosomal acidification and by preventing presentation of antigens to the immune system^2^. As the infection progresses, the lesions in the intestine and mesenteric lymph nodes become more severe. Rather than localized, the granulomatous infiltrate becomes diffuse disrupting the mucosal structure and function and affecting jejunum and ileum^3,4^. Although understanding the immunological mechanisms leading to the various outcomes of MAP infection remains problematic, there is no doubt that the interaction of Mycobacteria with the innate immune system plays a central role in the disease pathogenesis^5^.

PTB is responsible for significant economic losses in dairy herds worldwide due to decreased milk production, increased management costs and premature culling from clinical disease^6–9^. Furthermore, there is some evidence suggesting that MAP infection might be associated with Crohn’s disease (CD) in humans^10^. Several studies have demonstrated that more than 50% of the dairy cattle herds are positive for MAP antibodies in USA and in Europe and, therefore, bovine PTB can be considered endemic in these areas^11,12^. Commercial inactivated vaccines against bovine PTB are very successful in reducing MAP presence in feces and tissues and in increasing both milk production and cattle productive life in infected farms^13,14^. However, PTB vaccination with heat-killed inactivated vaccines is not allowed in most European countries due to its interference with *Mycobacterium bovis* detection tests^15^. Therefore, PTB control is currently based on testing and culling plus preventing MAP transmission to susceptible animals using appropriate hygienic-sanitary strategies^16,17^. However, the efficiency of the control programs based on the “test and cull” policy is strongly conditioned by the sensitivity of the tests used to identify early infections. Presently, fecal culture is considered “the gold standard” test for the ante-mortem diagnosis of MAP infection^18^. However, individual fecal culture is expensive and time consuming and only detects advanced infections due to the relatively late onset of fecal shedding during the natural course of MAP infection. In fact, the sensitivity of the fecal culture is 70% in animals with PTB-associated clinical signs, but only 23–29% in animals with subclinical infection, which may shed MAP intermittently and in lower numbers in feces and milk contaminating the environment and infecting other animals^19^. Early stage diagnostics such as the *IFN-γ* release assay detects whether a T-cell mediated immune response has been elicited in response to mycobacterial antigens but only reflects MAP exposure, and thus cannot discriminate between individuals with controlled infection from those with subclinical disease^20^. It is clear that the detection of subclinical infections before the bacterium is shed and transmitted to herd mates and possibly to humans remains a challenge and, therefore, novel tools are needed to detect MAP-infected animals at early stages of the infection.

Transcriptomic profiling is a promising source of novel biomarkers for early MAP infection in cattle, for monitoring the progress of the disease, and for the search of immune markers that can be used in the design of therapeutics and vaccines^21^. RNA-Sequencing (RNA-Seq) gives a quantitative and qualitative view of the transcriptomic profile of the tissues or cells being studied. Previous RNA-Seq analysis examined the transcriptome of bovine monocyte-derived macrophages (MDM) infected with MAP and revealed putative biomarkers for early infection^22,23^. To date only one study has used RNA-Seq to examine the transcriptome of ICV samples collected from dairy cows naturally infected with MAP^24^. Some of the subclinical animals included in this study did not show any specific evidence of MAP infection and no pathways controlling the immune response appeared to be affected in this animal group. The authors concluded that perhaps with deeper sequencing in further experiments, potential biomarkers could be ascertained. Since the peripheral blood (PB) is the most available physiological fluid for the detection of biomarkers, in the current study we examined whether it recapitulates, at least in part, the transcriptome of the ileocecal valve (ICV), the primary site of MAP colonization. For this purpose, RNA-Seq was used to identify host genes differentially expressed (DE) in ICV and PB samples collected from PTB-infected animals with focal or diffuse lesions in gut tissues versus control animals. Gene expression data were interpreted using gene ontology (GO) categories and metabolic pathways containing multiple DE genes. Overall, our study provides insights into how cattle respond to a natural MAP infection in two targets, ICV and PB, and revealed common and unique biological processes activated in animals with early and more advanced PTB-associated lesions.

## Results

### Summary of the RNA-Seq data

The animals included in our study were tested by multiple diagnostic tests including histopathological analysis of gut tissues, acid-fast stain, specific antibody response to MAP, fecal qPCR and tissue and fecal bacteriological culture. The results of the premortem (at the annual sampling time) and post-mortem (at the time of slaughter) diagnostic tests are showed in Table 1. All control animals (n = 3) showed a negative result for all diagnostic tests. Culling of control animals was due to age, accident, and coxofemoral luxation. All the infected cows included in our study had detectable lesions in gut tissues with distinct severity, focal or diffuse. At the slaughter time, the animals with focal lesions in gut tissues (n = 6) showed a negative fecal culture result but were fecal PCR positive (1/6), ELISA positive (1/6), and/or had evidence of MAP infection in gut tissues by ZN (1/6), PCR (5/6) and/or bacteriological culture (2/6). None of the animals with focal lesions had a heavy bacterial load (>50 cfu/gr) in feces and tissues and none of them was culled due to paratuberculosis-associated clinical signs. In contrast, the cows with diffuse histopathological lesions in gut tissues (n = 5) were culled due to a positive ELISA or PCR result (2/5), or because they showed PTB-associated clinical signs, including low milk production (1/5) or profuse diarrhea and weight loss (2/5). At the slaughter time, all of the cows with diffuse lesions had a positive ZN and ELISA result, a PCR-positive result in feces and tissue samples, and had a positive bacteriological culture from gut tissues. The mean amount of MAP estimated by qPCR in the gut tissues of all the animals with diffuse lesions was >66765 copies of MAP/gr tissue while the mean bacterial load in the gut tissue samples of the animals with focal lesions was ˂ 30528 copies of MAP/gr tissue.

**Table 1:** Histopathological analysis, ZN stain, ELISA, PCR and bacteriological culture results from all the animals included in the current study.

ID	Histopathological analysis			ELISA (OD)		Fecal PCR		Fecal culture (CFUs)		Gut tissues	
	Microscopic	Macroscopic	ZN	Pre-mortem	At slaughter	Pre-mortem	At slaughter	Pre-mortem	At slaughter	PCR	Culture
1	Negative	No	Neg	Neg (1.26)	Neg (1.93)	Neg	Neg	Neg	Neg	Neg	Neg
2	Negative	No	Neg	Neg (2.77)	Neg (8.54)	Neg	Neg	Neg	Neg	Neg	Neg
3	Negative	No	Neg	Neg (8.99)	Neg (2.45)	Neg	Neg	Neg	Neg	Neg	Neg
4	Focal	No	Neg	ND	Neg (2.71)	ND	Neg	ND	Neg	Pos (0.38)	Neg
5	Focal	No	Neg	Neg (1.00)	Neg (1.03)	Neg	Neg	Neg	Neg	Pos (7.50)	Low
6	Focal	No	Neg	Neg (4.39)	Neg (5.51)	Neg	Neg	Neg	Neg	Pos (305.28)	Neg
7	Focal	No	Neg	Neg (4.92)	Neg (0.74)	Pos (0.03)	Neg	Neg	Neg	Pos (12.26)	Neg
8	Focal	No	Neg	Pos (95.85)	Pos (133.17)	Neg	Pos	Neg	Neg	Pos (70.54)	Medium
9	Multifocal	Yes	Pos	Neg (0.00)	Neg (2.97)	Neg	Neg	Neg	Neg	Neg	Neg
10	Diffuse paucibacillary	Yes	Pos	Neg (53.00)	Pos (283.13)	Neg	Pos	Neg	Neg	Pos (667.65)	Heavy
11	Diffuse intermediate	Yes	Pos	Pos (142.31)	Pos (187.69)	Pos (0.14)	Pos (0.14)	Neg	Neg	Pos (3896.19)	Heavy
12	Diffuse intermediate	Yes	Pos	Pos (174.01)	Pos (241.18)	Pos (7.50)	Pos (114.00)	Neg	Heavy	Pos (104032)	Heavy
13	Diffuse intermediate	Yes	Pos	Neg (32.19)	Pos (288.75)	Pos (1.41)	Pos (39.88)	Neg	Neg	Pos (1572.24)	Low
14	Diffuse multibacillary	Yes	Pos	Pos (286.51)	Pos (255.55)	Pos (11.70)	Pos (2832.00)	Neg	Neg	Pos (173316.00)	Heavy

Neg, Negative; Pos, Positive; ZN, Ziehl-Neelsen; OD, Optical Density; CFU, Colony Forming Units; ND, No Determined. DNA samples with a PCR-positive result using the LSI VetMax Triplex real-time PCR were quantified using the ParaTB Kuanti-VK kit. qPCR results are expressed as MAP DNA copies per gram of feces or tissues × 10^2^.

RNA-Seq libraries were prepared from PB and ICV of 14 animals within the three categories; with focal or diffuse histopathological lesions in gut tissues and control. Duplicate RNA extractions and corresponding RNA-Seq libraries were generated from PB and ICV samples of five of the 14 cows (ID 2, 6, 8, 11, 12) to check the reproducibility of the RNA-Seq technology. The RNA-Seq data summary for each biological sample and experimental replicate including number of reads per individual RNA-Seq library at each phase of the analysis is provided in Supplementary Table I. All the libraries were sequenced generating an average of de 22.31 million raw reads per library with a Phred quality score >30 which is considered a benchmark for quality in next-generation sequencing. Following filtering of reads based on quality score, minimum size of 60 bp and ambiguous base N percentage less than 5%, an average of 21.44 million reads remained. Alignment of the filtered RNA-Seq reads to the *Bos taurus* reference genome yielded mean values per library of 19.96 million reads. From the mapped reads, an average of 5% of the reads mapped to multiple locations in the genome and were excluded for gene expression analysis. A more detailed analysis of the reads mapping to unique locations was obtained using bedtools and featureCounts, two software packages developed for counting reads to genomic features such as upstream and downstream regions of genes, exons, introns and intergenic regions. Analysis of the individual library reads from the PB samples revealed that 57% of the reads aligned to exons, 28% to introns, 3% to intergenic regions, 2% to upstream regions and 10% to downstream regions. In the ICV samples, introns accounted for 24% of reads, exons for 63% of the reads, 2% of the aligned reads mapped intergenic regions, 1% upstream regions and 10% downstream regions.

### Analysis of differential gene expression from RNA-Seq data

Although high technical reproducibility has been claimed for the RNA-Seq technology^25,26^, we performed duplicate RNA extractions and corresponding RNA-Seq library preparations from PB and ICV samples of some of the animals (ID 2, 6, 8, 11, 12). Variations in this experiment could arise from the RNA preparation, fragmentation or priming method, ligation efficiencies, amplification and other variables in the technical steps of library construction and amplification. After performing gene expression analysis as describe in materials and methods, no significant differences were observed between each sample and corresponding replicate which support the reproducibility of gene expression studies using RNA-Seq (Supplementary Fig. 1).

**Figure 1:**
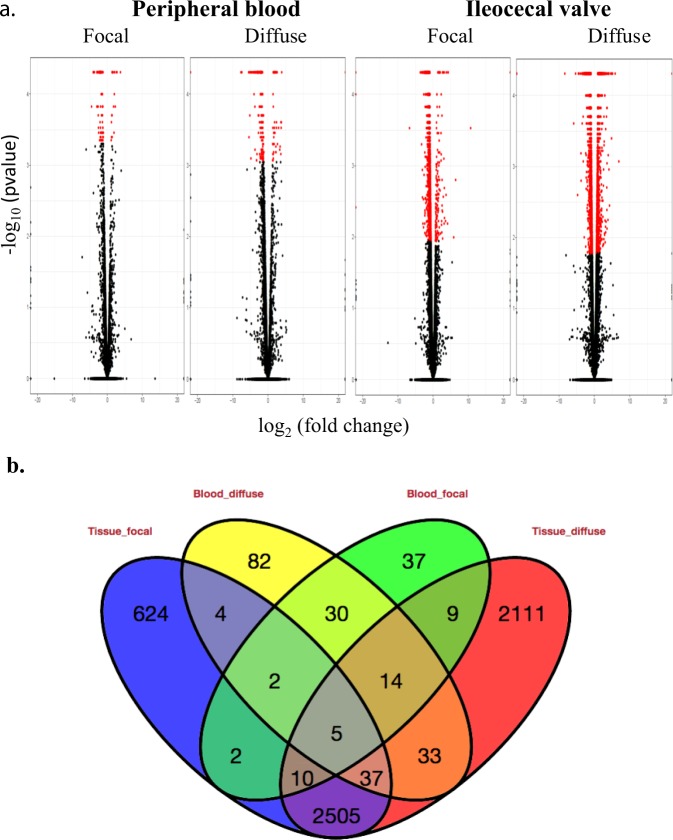
RNA-seq analysis. (**a**) Volcano plots showing the DE genes (log_2_ fold change) versus –log (p value) for each comparison. The red spots represent the DE genes in each comparison. (**b**) Venn diagram showing transcriptional changes between the groups comparisons based on RNA-Seq data.

The DE genes (FDR < 0.05) between cows with focal or diffuse histopathological lesions versus control cows are presented as red dots in Fig. 1a. The number of DE genes was higher in the ICV samples than in the PB samples and increased in animals with diffuse histopathological lesions when compared with the group of animals with focal lesions. This was expected since diffuse lesions represent a more advanced stage of the disease. In the PB samples, 109 and 207 genes were DE in the comparisons between animals with focal or diffuse lesions and control cows, respectively (Fig. 1b). Data analysis revealed 51 DE genes that were common in the PB samples regardless of the type of lesion. The transcriptomic analysis of the ICV samples showed that 3189 and 4724 genes were DE in the animals with focal or diffuse lesions when compared with control animals, respectively. Between these two comparisons, 2557 genes were DE in the ICV samples of the MAP-infected cows regardless of the comparison. A total of 19 DE genes were common among the animals with focal lesions regardless of the tested sample, ICV or PB. Similarly, 89 genes appeared DE in the PB and ICV samples of the animals with diffuse lesions.

### Gene expression comparison between animals with PTB-associated histophalogical lesions versus control cows

A total of 109 and 207 were DE in the PB samples of the animals with focal or diffuse lesions when compared with the control animals, respectively. Among these DE genes, 21 and 48 were upregulated in each comparison, respectively (Table 2). Table 3 shows the top five upregulated and top-five downregulated genes in the PB samples of the animals with focal lesions versus control samples ranked by fold-change. The top five upregulated genes included the *ATP binding cassette subfamily A member 13* (*ABCA13*), a receptor for class I MHC antigens named *Leukocyte immunoglobulin-like receptor subfamily A member 6* (*LOC618*4*16*) and three still uncharacterized proteins. The top five genes downregulated in the PB samples from the animals with focal lesions versus control cows were: the *C-C motif chemokine ligand 14* (*CCL14*), *Insulin-like growth factor 2 mRNA binding protein 3* (I*GF2BP3*), *Osteoglycin* (*OGN*), *Glycogen phosphorylase muscle associated* (*PYGM*), and the *CD5 antigen-like precursor* (*CD5L*). Many of the top ranked DE genes have immune-related functions such *LOC618416*, *CD5L* and *CCL14*; being the last two genes downregulated. The *CD5L* is a secreted protein expressed by macrophages in lymphoid and inflamed tissues that acts as a key regulator of lipid synthesis. *CCL14* is a chemotactic factor that attracts T-cells and monocytes, but nor neutrophils, eosinophils, or B-cells. Fold change in the downregulated genes ranged from −4.5 to −1.1, with a mean fold change of −2.0. For the upregulated genes, the fold change of expression ranged between 3.7 to 1.0 with a mean log fold of 1.7.

**Table 2:** Differentially expressed (DE) genes and specific DE genes for each comparison.

Sample	Comparison	DE genes (N)	Up-regulated genes (N)	Down-regulated genes (N)	Comparison specific DE genes (N)	Up-regulated genes (N)	Down-regulated genes (N)
PB	Focal vs Control	109	21	88	37	11	26
PB	Diffuse vs. Control	207	48	159	82	21	61
PB	Focal vs. Control + Diffuse vs. Control	51	6	45	30	4	26
ICV	Focal vs. Control	3189	168	3021	620	68	552
ICV	Diffuse vs. Control	4724	1473	3251	2105	1321	784
ICV	Focal vs. Control + Diffuse vs. Control	2557	96	2446	2505	93	2397
PB, ICV	Focal vs. Control	19	0	18	2	0	2
PB, ICV	Diffuse vs. Control	89	20	49	33	17	12
PB, ICV	Focal vs. Control + Diffuse vs. Control	5	0	4	5	0	4

**Table 3:** The top five up- and downregulated DE genes in samples from MAP-infected cows versus control samples.

Sample	Comparison	Gene ID	Description	Fold Change (Log_2_)^a^
PB	Focal vs. Control	ENSBTAG00000003531	*ATP binding cassette A member 13 (ABCA13)*	3.7
		ENSBTAG00000039691	Uncharacterized protein	2.7
		ENSBTAG00000039086	Uncharacterized protein	2.6
		ENSBTAG00000000930	Uncharacterized protein	2.2
		ENSBTAG00000019348	*leukocyte Ig-like receptor subfamily A member 6*	2.0
		ENSBTAG00000022514	*CD5 antigen-like precursor (CD5L)*	−3.6
		ENSBTAG00000001032	*Glycogen phosphorylase,muscle associated (PYGM)*	−3.8
		ENSBTAG00000011824	*Osteoglycin (OGN)*	−3.9
		ENSBTAG00000019406	*Insulin growth factor 2 binding protein 3 (IGF2BP3)*	−4.0
		ENSBTAG00000010738	*C-C motif chemokine ligand 14 (CCL14)*	−4.5
	Diffuse vs. Control	ENSBTAG00000046419	*Two pore channel 3 (TPC3)*	3.8
		ENSBTAG00000013921	*Creatine kinase, M-type (CKM)*	3.8
		ENSBTAG00000005353	*Desmin (DES)*	3.7
		ENSBTAG00000046611	Uncharacterized protein	3.6
		ENSBTAG00000018369	*Myosin light chain 2 (MYL2)*	3.6
		ENSBTAG00000047764	*multidrug resistance-associated protein 4-like*	−5.1
		ENSBTAG00000010738	*C-C motif chemokine ligand 14 (CCL14)*	−5.3
		ENSBTAG00000002976	*CD177 molecule (CD177)*	−5.4
		ENSBTAG00000031599	*Cathelicidin-3 (CATHL3)*	−7.4
		ENSBTAG00000020072	*Cathelicidin-4 (CATHL4)*	−7.7
ICV	Focal vs. Control	ENSBTAG00000022937	*Intelectin 2 precursor (ITNL2)*	10.6
		ENSBTAG00000013027	*Cholecystokinin (CCK)*	6.3
		ENSBTAG00000014663	*Homeobox B13 (HOXB13)*	5.8
		ENSBTAG00000010632	*Fatty acid binding protein 6 (FABP6)*	4.2
		ENSBTAG00000039035	*Heat shock protein 70 family A member 6 (HSPA6)*	4.2
		ENSBTAG00000020184	*Aquaporin 8 (AQP8)*	−3.2
		ENSBTAG00000019379	*Tumor suppressor candidate 5 (TUSC5)*	−3.2
		ENSBTAG00000046611	Uncharacterized protein	−3.6
		ENSBTAG00000013055	*Duodenase-1-like*	−3.6
		ENSBTAG00000047008	Uncharacterized protein	−6.7
	Diffuse vs. Control	ENSBTAG00000022937	*Intelectin 2 precursor (ITLN2)*	6.8
		ENSBTAG00000039035	*Heat shock protein family 70A member 6 (HSPA6)*	5.8
		ENSBTAG00000048075	*Ig superfamily member 23 (IGSF23)*	5.1
		ENSBTAG00000008505	*Apolipoprotein B (APOB)*	4.8
		ENSBTAG00000016662	*Carbamoyl-phosphate synthase 1 (CPS1)*	4.8
		ENSBTAG00000020184	*Aquaporin 8 (AQP8)*	−4.5
		ENSBTAG00000001417	*Acyl-CoA synthetase medium chain 1 (ACSM1)*	−4.7
		ENSBTAG00000026323	*Lysozyme C, intestinal isozyme (LYSB)*	−5.3
		ENSBTAG00000019813	*Adiponectin, C1Q (ADIPOQ)*	−5.8
		ENSBTAG00000011941	*Lysozyme C, milk isozyme (LYZ1)*	−8.3

^a^A negative fold-change value reports downregulated gene expression.

RNA-Seq analysis detected 207 DE genes in the PB samples of the cows with diffuse lesions compared to the control group. A total of 48 out of the 207 DE genes were upregulated (mean fold change of 2.1, range between 3.8 to 1.3) and 159 downregulated (mean fold change of −2.3, range between −1.1 to −7.7). The top five upregulated genes in the PB samples of the animals with diffuse lesions when compared with control animals were: *Two pore channel 3* (*TPC3*), *Creatine kinase M-type* (*CKM*), *Desmin* (*DES*), *Myosin light chain 2* (*MYL2*) and an uncharacterized protein (Table 3). The top five downregulated genes in this comparison were: the *Cathelicidins 3* and *4* (*CATHL3* and *CATHL4*), *CD177* molecule (*CD177*), *C-C motif Chemokine ligand 14* (*CCL14*), and the *Multidrug resistance-associated protein 4-like*. *CATHL3* and *CATHL4* exert a potent antimicrobial activity due to an impairment of the function of the respiratory chain and of energy-dependent activities in the inner membrane of susceptible microorganisms. *CD177* is a neutrophil G-protein-linked surface glycoprotein.

RNA-Seq analysis revealed that 3189 and 4724 genes were dysregulated in the ICV samples from the animals with focal or diffuse lesions versus de control group, respectively (Table 2). The top five upregulated genes in the ICV of cows with focal lesions were the *Intelectin 2* precursor (*ITLN2*), *Cholecystokinin* (*CCK*), *Homeobox B13* (*HOXB13*), *Fatty acid binding protein 6* (*FABP6*) and *Heat shock protein family A member 6* (*HSPA6*). The upregulated genes mean fold change of expression was 1.5 but, in this case there were larger differences in the expression levels between genes, having the most upregulated gene a fold change of 10.6 and the less upregulated one a fold change of 0.6. *CCK* is a peptide hormone that induces gall bladder contraction and release of pancreatic enzymes in the gut. *HOXB3* is a transcriptional factor previously implicated with inflammation. *FABP6* is involved in the gastric acid and pepsinogen secretion and is also required for efficient apical to basolateral transport of conjugated bile acids in ileal enterocytes. The top five downregulated genes were two uncharacterized proteins, the *Duodenase-1-like* with both trypsin‐like and chymotrypsin‐like activities, *Tumor suppressor candidate 5* (*TUSC5*), and the *Aquaporin 8* (*AQP8*) (Table 3). Mean fold change of the downregulated genes was −1.1 with values ranging between −6.7 and −0.6.

In the ICV samples, a total of 4724 DE genes were identified in the comparison between the animals with diffuse lesions versus the control group. From these 4724 DE genes, 1473 genes were upregulated and 3251 were downregulated (Table 2). The level of gene expression was similar between the up- and downregulated genes as demonstrated by the log_2_ fold changes. The upregulated genes on average showed a 1.2 fold increase between the cows with diffuse lesions and the control group (range between 6.8 to 0.5), whereas the downregulated genes averaged a −1.2 fold decrease between groups (range between −8.3 to −0.5). The top five upregulated genes included the *Intelectin 2* precursor (*ITLN2*), *Heat shock protein family A member 6* (*HSPA6*), *Immunoglobulin superfamily member 23* (*IGSF23*), *Apolipoprotein B* (*APOB*) and the *Carbamoyl-phosphate synthase 1* (*CPS1*). On the other hand, the top five downregulated genes were the *Lysozyme C milk isozyme 1* (*LYZ1*), *Adiponectin C1Q* (*ADIPOQ*), *Lysozyme C intestinal isozyme* (*LYSB*), *Acyl-CoA synthetase medium chain family member 1* (*ACSM1*) and the *Aquaporin 8* (*AQP8*). Several of the downregulated genes in this comparison such as the *ITLN2, LYZ1, LYSB* and *ADIPOQ* play important roles in the defense response against pathogens. Lysozymes such as *LYZ1* and *LYSB* have primarily a bacteriolytic function. *ADIPOQ* is an adiponectin involved in the control of the fat metabolism and it has an anti-inflammatory activity by negatively regulating *TNF-α* expression in macrophages, and also by counteracting its effects.

### Common gene expression signatures in the PB and ICV samples of MAP-infected animals

DE gene analysis revealed that 51 genes were DE in the PB of the MAP-infected animals regardless of the severity of the lesion (Table 2). Similarly, 2557 genes were DE in the ICV gene expression profiles from the infected cows versus the control group. The PB and ICV gene expression profiles from the cows with focal lesions shared 19 DE genes, and 89 DE genes were common in the PB and ICV expression profiles of the cows with diffuse lesions. In Table 4 we show the top three upregulated and downregulated genes in the PB and/or ICV gene expression profiles from MAP-infected animals. The *Oxidized low density lipoprotein receptor* (*ORL1*) and the *Tweety family protein 2* (*TTYH2*) appeared both upregulated in the PB samples of the MAP-infected cows, regardless of the severity of their lesions in gut tissues. The *CCL14* chemokine, an epithelial-derived chemokine with antibacterial properties, was the most downregulated gene in the PB gene expression profile from MAP-infected cows versus the control group.

**Table 4:** The top 3 up- and downregulated genes in the PB and/or ICV samples of infected animals with focal and/or diffuse lesions versus control samples.

Sample	Comparison	Gen ID	Description (Gene Symbol)	Lesion	Fold Change (Log2)^a^
PB	Focal vs. Control + Diffuse vs. Control	ENSBTAG00000019348	Uncharacterized protein	Focal	2.0
				Diffuse	1.6
		ENSBTAG00000004547	*Oxidized low density lipoprotein receptor1 (OLR1)*	Focal	1.1
				Diffuse	2.3
		ENSBTAG00000011007	*Tweety family member 2 (TTYH2)*	Focal	1.3
				Diffuse	1.7
		ENSBTAG00000001032	*Glycogen phosphorylase (PYGM)*	Focal	−3.8
				Diffuse	−2.7
		ENSBTAG00000019406	*Insulin growth factor2 mRNA binding prot3 (IGF2BP3)*	Focal	−4.0
				Diffuse	−2.8
		ENSBTAG00000010738	*C-C motif chemokine ligand 14 (CCL14)*	Focal	−4.5
				Diffuse	−5.3
ICV	Focal vs. Control + Diffuse vs. Control	ENSBTAG00000022937	*Intelectin 2 precursor (ITLN2)*	Focal	10.6
				Diffuse	6.8
		ENSBTAG00000010632	*Fatty acid binding protein 6 (FABP6)*	Focal	4.2
				Diffuse	4.7
		ENSBTAG00000039035	*Heat shock protein family A member 6 (HSPA6)*	Focal	4.2
				Diffuse	5.8
		ENSBTAG00000047181	*Multidrug resistance-associated protein 4*	Focal	−3.2
				Diffuse	−2.3
		ENSBTAG00000020184	*Aquaporin 8 (AQP8)*	Focal	−3.2
				Diffuse	−4.5
		ENSBTAG00000013055	*Duodenase-1-like*	Focal	−3.6
				Diffuse	−2.0
PB + ICV	Focal vs. Control	ENSBTAG00000036154	*TCR gamma alternate reading frame protein (TARP)*	Blood	−2.8
				ICV	−1.1
		ENSBTAG00000002362	*Apolipoprotein L domain containing 1 (APOLD1)*	Blood	−2.6
				ICV	−1.1
		ENSBTAG00000038080	*Duodenase-1*	Blood	−2.2
				ICV	−1.9
	Diffuse vs. Control	ENSBTAG00000011467	*Basic ATF transcription factor2 (BATF2)*	Blood	2.6
				ICV	2.8
		ENSBTAG00000001725	*C-X-C motif chemokine ligand 10 (CXCL10)*	Blood	2.0
				ICV	2.2
		ENSBTAG00000046803	*Growth arrest specific 1 (GAS1)*	Blood	2.4
				ICV	1.1
		ENSBTAG00000039196	*C4b-binding protein alpha-like*	Blood	−4.8
				ICV	−2.0
		ENSBTAG00000010273	*Epiregulin (EREG)*	Blood	−4.8
				ICV	−2.0
		ENSBTAG00000019716	*C-X-C motif chemokine ligand 8 (CXCL8)*	Blood	−3.9
				ICV	−1.9

^a^A negative fold-change value reports downregulated gene expression.

**Figure 2:**
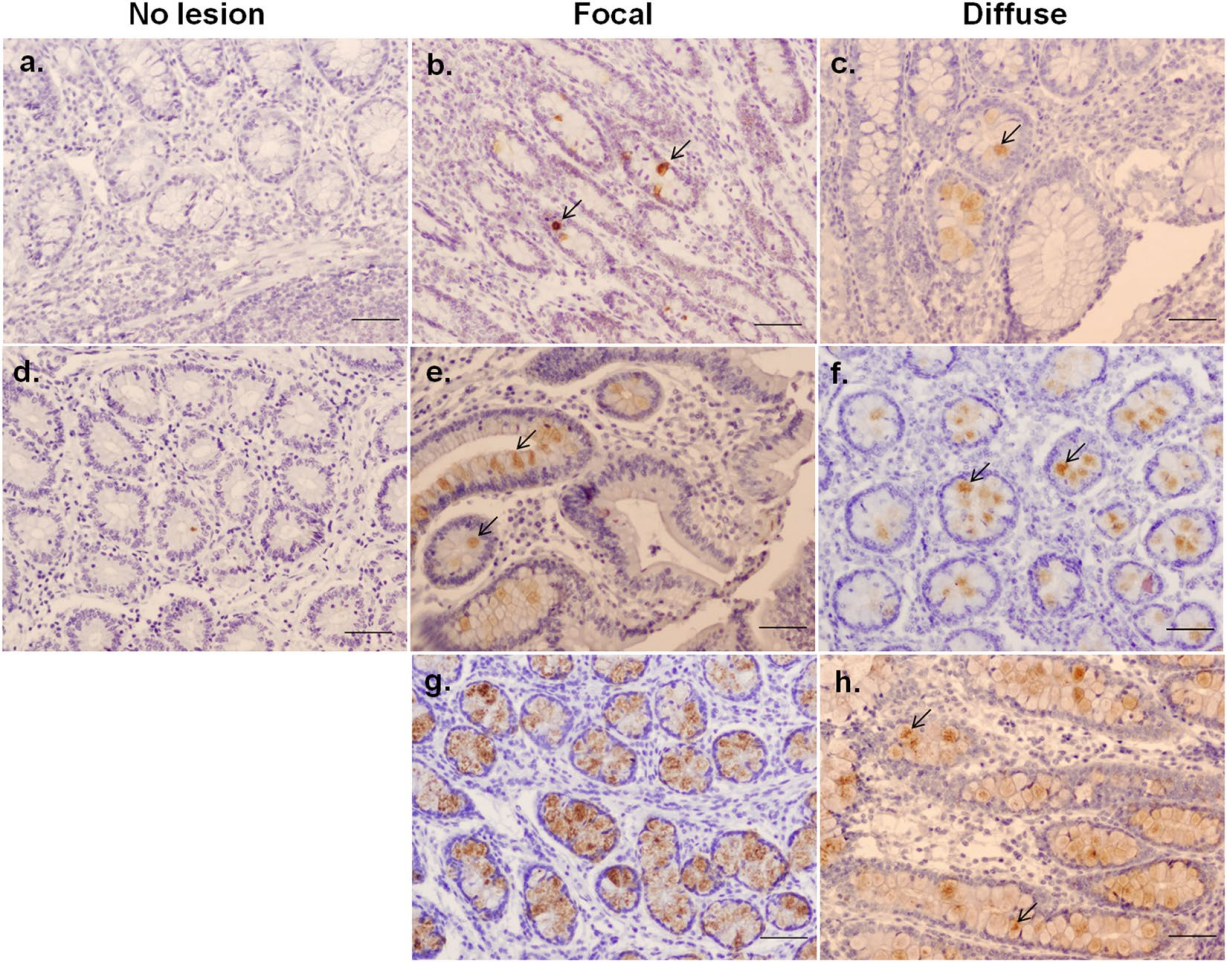
Localization of the *Intelectin 2* (*ITLN2*) precursor in the ICV of MAP-infected versus control cows by immunohistochemistry. Sections of the ICV from animal without lesions (**a,d**), and with focal (**b,e,g**) or diffuse lesions (**c,f,h**) were labeled with an anti-bovine ITLN2 antibody and stained as explained in materials and methods. The arrows show sites of antibody binding. Bars are 50 µM.

In the ICV samples, the *ITLN2* precursor showed the highest transcription increase in the cows with both focal and diffuse lesions; log_2_ fold-change was 10.6 and 6.8, respectively. Immunohistochemistry provided further evidence of the *ITLN2* production and localization in ICV. As seen in Fig. 2, *ITLN2* expression increases upon infection with MAP compared to control samples. Goblet cells and Paneth cells along the ICV from the infected animals were intensively labeled with the bovine *ITLN2* antibody. *FABP6*, a gastrotropin involved in the efficient apical to basolateral transport of conjugated bile acids in ileal enterocytes was highly upregulated in the ICV gene expression profile from the infected cows irrespective of the severity of the histopathological lesions. The stress response protein, *HSPA6*, was also highly expressed in the ICV from MAP-infected cows with log_2_ fold-changes of 4.2 and 5.8 in the gene expression profiles of the cows with focal or diffuse lesions versus control cows, respectively. A significant reduction in the expression of the *AQP8* gene, a protein involved in water/solute intestine homeostasis, was observed in the ICV gene expression profile from the cows with focal or diffuse lesions versus the control group; log_2_ fold-change = −3.2 and −4.5, respectively.

Several genes involved in the control of infectious diseases such as the *Basic leucine zipper ATF transcription factor 2* (*BATF2*) and the *C-X-C motif chemokine ligand 10* (*CXCL10*), were upregulated in the PB and ICV gene expression profiles from the animal with diffuse lesions. *BATF2* belongs to the activator protein 1 (*AP-1*) transcription factor family and interacts with the *IFN regulatory factor 1* to mediate downstream proinflammatory immune responses. *CXCL10* is chemotactic for monocytes and T-lymphocytes by binding to *CXCR3*. *C4b-binding protein alpha-like*, *epiregulin* (*ERG*) and the *CXCL8/IL8* were downregulated in the PB and ICV gene expression profiles from cows with diffuse lesions versus the control group.

Five genes were DE in the PB and ICV gene expression profiles from the animals with PTB-associated histopathological lesions irrespective of the type of lesion including the *CXCL8/IL8*, *Apolipoprotein L domain containing 1 (APOLD1*), *Interferon α-inducible protein 27* (*IFI27*), *KIAA1324 like* (*KIAA1324L*) and *ArfGAP with RhoGAP domain, ankyrin repeat and PH domain 2* (*ARAP2*). *CXCL8/IL8*, *APOLD1*, *KIAA1324L* and *ARAP2* were downregulated in all the comparisons. The *ARAP2* gene is thought to play a major role in the activation of the *major histocompatibility complex class II* genes expression^27^. The *IFI27* was downregulated in the PB samples and upregulated in the ICV samples from the cows with focal (log_2_ fold = 1.6) and diffuse lesions (log_2_ fold = 2.6) when compared with the control group. The *IFI27* (*ISG12*) mediates *IFN*-induced apoptosis characterized by a rapid and robust release of cytochrome C from the mitochondria and activation of caspases 2,3,6,8 and 9 which may in turn influence the antiviral activities of the *IFN*^28^.

### Gene ontology (GO) analysis

**Figure 3:**
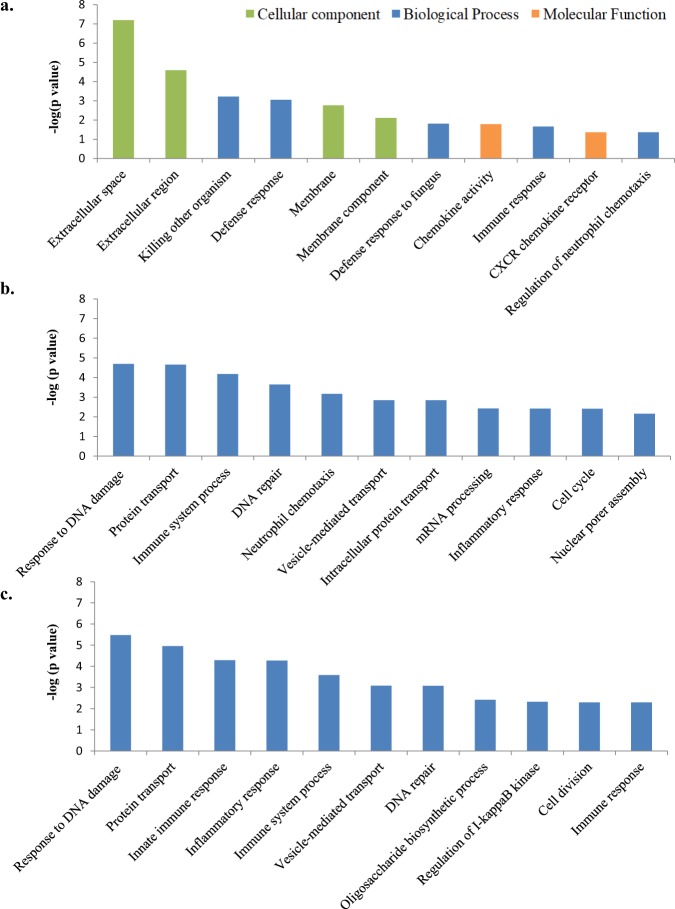
Gene ontology (GO) analysis using the DE genes in the two groups of infected cows compared to the control group. (**a**) The eleven GO enriched in the PB samples collected from the group of animals with diffuse lesions in gut tissues versus the control group. (**b**) The 11 top biological processes overrepresented in the ICV samples collected from the animals with focal lesions versus the control group. (**c**) The 11 top biological processes enriched in the ICV samples from the group of cows with diffuse lesions versus the control group.

Functional categorization of the DE genes in each comparison was performed using the Bioconductor GOseq package to identify biological process (BP), cellular component (CC) and molecular function (MF) (Table 5). While in the PB samples of the cows with focal lesions we did not identify any significantly overexpressed GO, eleven GOs were significantly enriched in the PB samples of the animals with diffuse lesions. Out of these eleven GO; 5 were BP, 4 were CC and 2 were MF (Fig. 3a). The five identified BP were related to the immune response, including killing of cells of other organisms (GO:0031640), defense response (GO:0006952, GO:0050832), immune response (GO:0006955) and positive regulation of neutrophil chemotaxis (GO:0090023). The enriched CC identified in the PB samples of cows with diffuse lesions were the extracellular space and cell membrane.

**Table 5:** Number of enriched gene ontologies (GOs) and specific GOs for each comparison.

Sample	Comparison	Total enriched GO (N)	Enriched Biological process (N)	Enriched Cellular component (N)	Enriched Molecular function (N)	Specific enriched GO (N)	Enriched Biological process (N)	Enriched Cellular component (N)	Enriched Molecular function (N)
PB	Focal vs. Control	0	0	0	0	0	0	0	0
PB	Diffuse vs. Control	11	5	4	2	7	3	2	2
PB	Focal vs. Control + Diffuse vs. Control	0	0	0	0	0	0	0	0
ICV	Focal vs. Control	83	32	31	19	34	13	12	9
ICV	Diffuse vs. Control	80	38	27	15	30	17	9	4
ICV	Focal vs. Control + Diffuse vs. Control	48	19	18	11	47	19	17	11
PB, ICV	Focal vs. Control	0	0	0	0	0	0	0	0
PB, ICV	Diffuse vs. Control	3	2	1	0	2	2	0	0
PB, ICV	Focal vs. Control + Diffuse vs. Control	0	0	0	0	0	0	0	0

**Figure 4:**
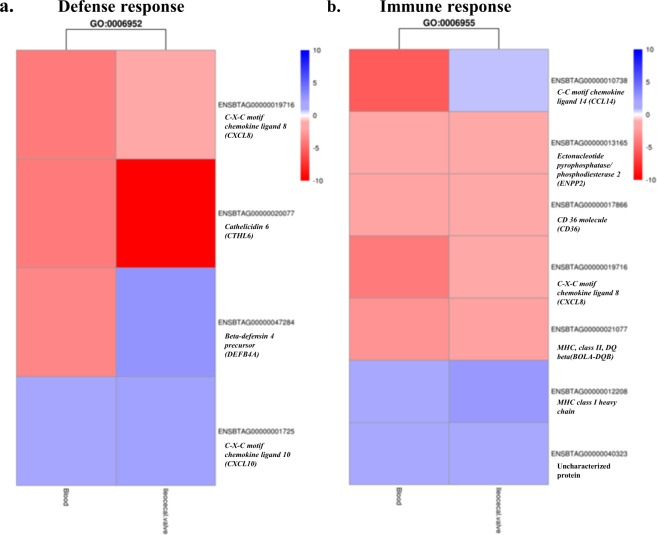
Enriched biological process in PB and ICV from the cows with diffuse lesions versus the control group. The defense response (GO:0006952) and immune response (GO:0006955) biological processes were both enriched in the PB and ICV gene expression profiles from the cows with diffuse lesions. The heatmaps represent the fold change (log_2_ fold) of the up- and downregulated genes included in the enriched GO:0006952 and GO:0006955. Colors and intensities depend on expression levels. Red indicated gene downregulation and blue upregulation.

We identified 83 and 80 overrepresented GO in the ICV samples of the cows with focal and diffuse lesions, respectively. The top 11 GOs identified in the ICV samples of the animals with focal and diffuse lesions were CC. Enriched CC in the ICV samples of the cows with both focal and diffuse lesions included the cytosol and the cytoplasm being these the top dysregulated CC. Among the top five BP enriched in the ICV samples of the cows with focal lesions were the cellular response to DNA damage stimulus (GO:0006974), protein transport (GO:0015031), immune system process (GO:00023746), DNA repair (GO:0006281) and the neutrophil chemotaxis GO (GO:0030593) (Fig. 3b). Three of the top five overexpressed BP in the ICV from the cows with diffuse lesions were associated with the innate immune and inflammatory response (Fig. 3c). Interestingly, the defense response (GO:0006952) and immune response (GO:0006955), were both enriched in the PB and ICV gene expression profiles from the cows with diffuse lesions (Fig. 4). Our results revealed that the chemokines *CXCL8/IL8* and *CXCL10* were regulated in opposite directions in cows with the more advanced lesions. While *CXCL8/IL8* was downregulated in the PB and ICV samples, *CXCL10* was upregulated in both MAP-targeted samples from the cows with diffuse lesions. This differential modulation of *CXCL10* and *CXCL8/IL8* may suggest a different role of both chemokines in the infection. While *CXCL8/IL8* attracts neutrophils, basophils and T-cells to the site of the infection, *CXCL10* is chemotactic for monocytes and T-lymphocytes by binding to CXCR3. Expression changes in genes with antibacterial activity such as the *Cathelicidin 6* (*CATHL6*) and *β-defensin* (*DEFβ4A*) were also observed. Seven DE genes included in the immune response pathway (GO:0006955) were dysregulated in the PB and ICV gene expression profiles from the cows with diffuse lesions including the *CCL14*, *ENPP2*, *CD36*, *CXCL8/IL8*, *BOLA-DQβ*, *MHC class I heavy chain* and one uncharacterized protein (ENSBTAG00000040323). While *MHC class I heavy chain* was upregulated; *ENPP2*, *CD36*, *CXCL8/IL8*, and *BOLA-DQβ* were downregulated in the PB and ICV expression profiles from the cows with diffuse lesions when compared with the control group.

### Metabolic analysis

Enriched metabolic routes related to the DE genes in each comparison were analyzed. Although no metabolic pathways referenced in the KEGG database were enriched in the PB samples, 78 and 82 metabolic routes were dysregulated in the ICV samples of the cows with focal and diffuse lesions, respectively. Only three of the 78 metabolic pathways dysregulated in the ICV gene expression profiles from the cows with focal lesions were significantly enriched including the N-Glycan biosynthesis (bta00510, P = 0.004), purine metabolism (bta00230, P = 0.015) and the one carbon pool by folate pathway (bta00670, P = 0.021). The metabolic analysis using the DE genes in the ICV from cows with diffuse lesions versus the control group revealed that the DE genes in this comparison affected metabolic processes such as the valine, leucine and isoleucine degradation (bta00280, P = 0.008) and the purine metabolism (bta00230, P = 0.003) witch sequences corresponding to 10 and 34 enzymes, respectively. Therefore, our metabolic analysis revealed bta00230 as a common metabolic route significantly enriched in the ICV from the infected cows versus the control cows. Using the STRING database, the bile secretion metabolic route (bta04976) was significantly enriched in the ICV from the cows with focal lesions (enrichment score = 244.99, FDR = 0.001) with 18 proteins involved in this network. In the ICV from the cows with diffuse lesions, the vitamin digestion and absorption (enrichment score = 599.07, FDR = 0.001) and the cholesterol (enrichment score = 677.38, FDR = 0.001) routes were significantly enriched with six and four proteins matching each route, respectively.

### Protein to protein interaction analysis

**Figure 5:**
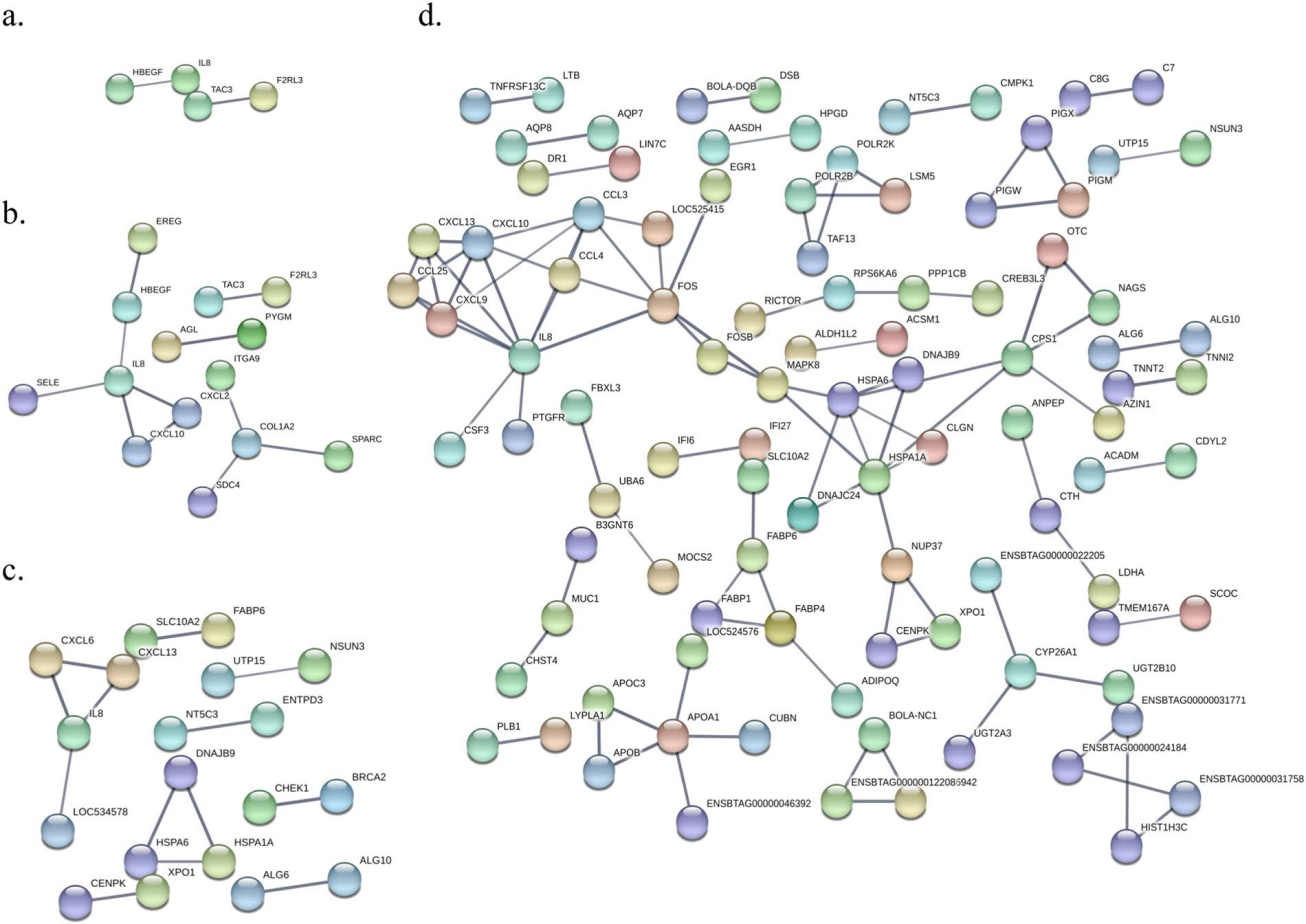
Protein-protein network analysis using the DE genes (log_2_ fold > 2 and < 2) in the transcriptomic profiles of the cows with PTB-associated lesions versus the control group. (**a**) Using the DE genes in the PB samples from the animals with focal lesions, two *HBEGF-IL8* and *TAC3-F2RL3* functional interactions were detected. (**b**) In the PB samples from the cows with diffuse lesions, two *IL8* and *COL1A2* centered networks were observed. (**c**) When the DE genes in the ICV transcriptomic profiles of the cows with focal lesions were used, a *IL8* centered network was also obtained. (**d**) A *MAPK8* centered network was retrieved when the DE genes in the ICV transcriptomic profile of the cows with diffuse lesions were searched in the STRING database. Individual nodes represent proteins with relationships represented by edges. The confidence score (>0.7) of each interaction is mapped to the edge thickness. The proteins with no associations to other proteins in the networks were hidden.

Protein to protein interaction analysis using the DE genes in the PB samples from the animals with focal lesions revealed a *HBEGF*-*CXCL8/IL8* functional association (Fig. 5a). Using the DE genes in the PB samples from the cows with diffuse lesions, 11 functional associations and two *CXCL8/IL8* and *Collagen type I, α2 chain* (*COL1A2*) networks were identified (Fig. 5b). The *COL1A2* was linked to a secreted protein, the *Acidic and rich in cysteine protein* (*SPARC*), the *Syndecan 4* (*SDC4*) and to *the Integrin subunit α9* (*ITGA9*). When the DE genes in the ICV gene expression profile from the cows with focal lesions were used, 13 functional interactions and one *CXCL8/IL8* centered network were retrieved (Fig. 5c). With a confidence cuff of 0.7, 90 functional interactions and a centered mitogen-activated *MAP kinase 8* (*MAPK8*) network were retrieved when the DE genes in the ICV of the animals with diffuse lesions were used (Fig. 5d). Our protein-protein interaction analysis revealed that the interaction between *MAPK8* and *CXCL8/IL8* might be mediated by two leucine zipper protein members of the AP1 transcription factor complex, *FOS* and *FOSB*.

## Discussion

Transcriptomic profiling of the host response to MAP infection has the potential to advance our understanding of MAP-host interactions and to reveal the mechanisms involved in the establishment and progression of bovine PTB. This information can help to set up the basis for novel diagnosis tools and for the development of novel preventive and therapeutic approaches which could improve cattle health. Host transcriptomic studies have highlighted the importance to compare whole blood versus other issues. To our knowledge, our study represents the first attempt to study the specificities and common signatures of the ICV and PB gene expression profiles of PTB-infected cattle using RNA-Seq. All the infected cows included in our study had detectable lesions in gut tissues with distinct severity, focal or diffuse. Although the number of control animals is limited, our results showed extensive expression differences between infected and control cows. We found that the number of DE genes was higher in the ICV than in the PB samples and increased with the severity of the lesion. More specifically, our study revealed 19 genes DE in the PB and ICV gene expression profiles from the cows with focal lesions versus the control group. Similarly, 89 genes were DE in the PB and ICV gene expression profiles from the cows with diffuse lesions versus the control group. Interestingly, the most upregulated gene in the ICV samples of the infected animals was the *ITLN2* precursor; log_2_ fold change = 10.6 and 6.8 in the cows with focal or diffuse lesions versus the control group, respectively. The *ITLN2* is an extracellular lectin able to bind *Mycobacterium tuberculosis* and *Mycobacterium bovis* bacillus Calmette-Guerin (BCG)^29^. The *FABP6*, an ileum-specific bile acid (BA) transporter, was also highly upregulated in the ICV gene expression profile from the infected cows versus control cows which suggests a dysregulation of the BA secretion in MAP-infected animals. We also observed that MAP infection downregulated the expression of the BA receptor FXR in the ICV from the cows with focal and diffuse lesions versus control cows; log_2_ fold = −0.8 and −1.0, respectively. In agreement with our results, Hempel *et al*. found the *FABP6* transporter as the top upregulated gene in the comparison of clinical versus uninfected control animals^24^. Apart from *FABP6*, we found other genes highly dysregulated in the ICV gene expression profile from the cows with diffuse lesions such as the *Immunoglobulin superfamily member 23* (*IGSF23*) (log_2_ fold = 5.1), *Apolipoprotein B* (*APOB*) (log_2_ fold = 4.8), *Solute carrier family 10 member 2* (*SLC10A2*) (log_2_ fold = 2.7), and the *Matrix metallopeptidase 13* (*MMP13*) (log_2_ fold = 2.0) which had been previously reported by Hempel *et al*. in the comparison of clinical versus control cows. Little is known about the role of *IGSF23*, but other *IGSF* members are cell adhesion molecules that perform important immunological functions, including recognizing a variety of counterpart molecules on the cell surface or extracellular matrix^30^.

Several genes involved in the control of infectious diseases such as the *basic leucine zipper transcription factor 2* (*BATF2*) and *CXCL10* chemokine were found in our study highly upregulated in the PB and ICV gene expression profiles from the cows with diffuse lesions versus the control group but were unaffected in the cows with focal lesions. *BATF2* is predominantly expressed in monocytes and macrophages and has a central role in macrophage activation by regulating inflammatory responses during mycobacterial infection. In human tuberculosis (TB), *BAFT2* has been suggested as a biomarker and a potential host directed drug target^31,32^. The *CXCL10* chemokine, also known as *IFN-γ inducible protein-10* (*IP-10*), is chemotactic for monocytes and T-lymphocytes by binding to CXCR3 receptor. *CXCL10* is expressed mainly by activated CD14+ memory T cells, which produce a Th1 pattern of cytokine production^33^. Several studies have demonstrated that *CXCL10* can be used as a biomarker of TB, where significantly greater levels of mycobacterial antigen-induced *CXCL10* protein were detected in whole blood of TB patients compared to healthy controls^34–36^. More recently, the simultaneously measurement of *CXCL10* and *IFN-γ* enhanced test sensitivity for bovine TB identification in cattle in clinical stages of the infection^37^.

Our study revealed enrichment of the following GOs in the ICV from the cows with focal lesions: cellular response to DNA damage stimulus (GO:0006974), protein transport (GO:0015031), immune system process (GO:0002376), DNA repair (GO:0006281) and neutrophil chemotaxis (GO:0030593). Three of the top five GO enriched in the ICV gene expression profile from the cows with diffuse lesions were associated with the innate immune response (GO:0045087), inflammatory response (GO:0006954) and the immune system process (GO:0002376). Interestingly, the GOs related to the defense response (GO:0006952) and immune response (GO:0006955) were enriched in the PB and ICV from the cows with diffuse lesions. The DE genes included in the GO:0006952 were the *CXCL8/IL8*, *CXCL10*, *CATHL6*, and *DEFβ4A*. Seven DE genes corresponding to the immune response (GO:0006955) were dysregulated in both the PB and ICV gene expression profiles from the cows with diffuse lesions including *CCL14*, *ENPP2*, *CD36*, *CXCL8/IL8*, *BOLA-DQβ*, *MHC class I heavy chain* and the uncharacterized protein ENSBTAG00000040323. *ENPP2*, *CD36*, *CXCL8/IL8*, and *BOLA-DQβ* were downregulated in the PB and ICV from the cows with diffuse lesions when compared with the control group. Downregulation of members of the bovine *major histocompatibility (MHC) class II* genes can potentially alter antigen presentation and activation of T helper cells during MAP infection^38^. Our results showed reduced expression of *BOLA-DOβ* and *BOLA-DQB* genes in the PB from the cows with focal and diffuse lesions, respectively. In the ICV samples, the *BOLA-DRα*, *BOLA-DMS*, *BOLA3*, and the *BOLA-DOB* genes were downregulated in cows with focal lesions when compared with the control group. In contrast, in the ICV samples of the cows with diffuse lesions upregulation of the *BOLA-DOα*, *BOLA-DMB*, *BOLA-DMA* genes was detected which might suggest the activation of T helper cells as the infection progresses.

Interestingly, our study revealed *CXCL8/IL8* as a common chemokine highly downregulated in our database and involved in the following pathways; neutrophil chemotaxis (GO:0030593), defense response (GO:0006952) and immune response (GO:0006955). Similarly, *CXCL8/IL8* production was reduced in stimulated PB mononuclear cells from CD patients in comparison to healthy subjects and in MDM from *Mycobacterium bovis*-infected cattle versus healthy control cattle^39,40^. Since the *CXCL8/IL8* is the major chemokine responsible for neutrophil recruitment and activation by binding to CXCR3, decreased *CXCL8/IL8* expression may reflect impairment of neutrophil recruitment and activation during a natural MAP infection. Other *CXCL8/IL8*-associated biological functions include plasma exudation, general granulocytophilia, degranulation of neutrophils, respiratory burst response, and mobilization of intra-cellular Ca^2+^ ^41^. In our study, the *Epiregulin* (*ERG*) and *CXCL8/IL8* genes were both downregulated in the PB and ICV gene expression profiles from the cows with diffuse lesions versus control cows. *ERG* belongs to the *epidermal growth factor* (*EGF*) family together with the *HBEGF*; acting both as mitogenic stimulators via binding to *EGF* receptors (*EGFR*s). Transactivation of *EGFR* leads to downstream signaling events including *MAPK8* phosphorylation and activation which leads to apoptosis induction^42^. Induced *CXCL8/IL8* release is predominantly dependent on *EGF* binding to the *EFGR* leading to activation of *MAPK8*^43^. Our protein-protein interaction analysis using the DE genes in the cows with diffuse lesions revealed a central role for *CXCL8/IL8* and *MAPK8* in MAP pathogenesis. This kinase was downregulated in the ICV gene expression profiles from the cows with focal and diffuse lesions when compared with the control group; −1.7 and −2.16938, respectively. Our results support the idea that MAP infection limits signaling via the *MAPK8-IL8* expression pathway which may favor MAP survival. Although the role of *CXCL8/IL8* in MAP pathogenesis has not been characterized before, the *CXCL8/IL8* has been shown to play an important role in the pathogenesis of the human inflammatory bowel disease (IBD)^33^.

Our protein-protein interaction analysis using the DE genes in the PB from the cows with focal and diffuse lesions identified a *CXCL8/IL8* network in both stages of the disease. In other hand, using the DE genes in the PB from the cows with diffuse lesions a *COL1A2* centered network was identified containing a *COL1A2-SPARC* functional interaction. Interestingly, a *SPARC*-centered network was expressed more strongly in *Mycobacterium bovis*-challenged MDM from bovine TB infected cows that in uninfected cows^44^. *SPARC*, also known as osteonectin, is a matrix protein that binds collagen, and it is required for the development of granuloma-like structures during chronic infections^45^. In our study, *SPARC* and *COL1A2* were upregulated in the PB gene expression profiles from the cows with diffuse lesions which suggests that the expression of both proteins could lead to a bad prognosis in MAP-infected cows.

Recently, metabolomic analysis yielded a clear separation between non-infected and MAP-infected cattle, indicating changes in general metabolism, nutrition uptake and energy balance during the early stage of the disease^46^. Using RNA-Seq, we identified three metabolic pathways significantly enriched in the ICV from the cows with focal lesions including the N-Glycan biosynthesis (map00510, P = 0.004), purine metabolism (map00230, P = 0.015) and the one carbon pool by folate route (map00670, P = 0.021). Altered concentrations of N-glycans, such as mannose, were previously observed by the Buck *et al*. early after MAP infection^46^. In addition, it has been recently proposed that *Mycobacterium tuberculosis* infection manipulates the glycosylation machinery and the N-Glycoproteome of human macrophages^47^. The metabolic analysis performed using the DE genes in the ICV from the cows with diffuse lesions revealed enrichment of the branched amino acids (BCAA; valine, leucine and isoleucine) degradation (map00280, p = 0.008) and of the purine metabolism (map00230, p = 0.003). The degradation of the BCAA in advanced stages of the infection is consistent with the fact that PTB eventually leads to intestinal malabsorption and hypoproteinemia in the final stages of the infection^4^. Interestingly, our study identified the purine metabolism as a common enriched route in the ICV gene expression profiles of MAP-infected cows regardless of the type of lesion. In agreement with our findings, a previous study showed that MAP infection increased intracellular ATP in bovine monocytes^48^. Using the STRING database, the bile secretion metabolic route (bta04976) was significantly enriched in the ICV gene expression profiles from the cows with focal lesion (enrichment score = 244.99, FDR = 0.001). In the ICV gene expression profile from the cows with diffuse lesions, the cholesterol and the vitamin digestion and absorption routes were significantly enriched, respectively. In agreement with these findings, recent reports have demonstrated that MAP is able to manipulate the host lipid metabolism and accumulate cholesterol within macrophages which might favor MAP persistence^49^. In addition, decreases in serum 25-hydroxyvitamin D_3_ (25OHD_3_) levels were significantly lower in cows in the clinical stage of disease compared with either cows in the subclinical stage and non-infected control cows^50^. It is generally accepted that cattle in advanced stages of MAP infection are unable to metabolize and absorb vitamin D_3_ across the intestinal wall, and therefore suffer from weight loss and cachexia.

This study is the first to simultaneously describe transcriptomic changes in PB and ICV samples of cattle naturally infected with MAP. Several important observations were made. First, by comparing the transcriptomic profiles of two MAP-targeted tissues we described both unique and overlapping changes in the transcriptome of the infected cows versus the control group. Second, our study highlighted the ability of the RNA-Seq technology to reveal roles for genes that have not been previously implicated in the host response to MAP infection. Third, our transcriptomic analysis provided potential biomarkers for the development of future diagnostic tools, vaccines and therapeutics.

## Materials and Methods

### Ethic statement

Experimental procedures performed on the animals used in this study were approved by the Animal Ethics Committee of the Servicio Regional de Investigation y Desarrollo Agroalimentario (SERIDA) and authorized by the Regional Consejeria de Agroganadería y Recursos Autoctonos of the Principality of Asturias (approval code PROAE 29/2015). All the procedures were carried out in accordance with the European Guidelines for the Care and Use of Animals for Research Purposes (2012/63/EU). PB and fecal samples were collected by trained personnel and in accordance with good veterinary practice.

### Animal population

Fourteen Holstein Friesian cows from a single commercial dairy farm in Asturias (Spain) were monitored annually by obtaining PB and fecal samples to assess disease status by ELISA, and fecal bacteriological culture and PCR. The mean prevalence of the disease in the farm estimated by ELISA was 6.29% in the sampling period (2016–2018).

### Tissue and fecal sampling for histopathology, immunohistochemistry and bacterial culture

For histopathological analysis, samples from ileocecal lymph nodes, distal jejunal lymph node, ICV, and distal jejunum were collected aseptically from each animal and placed in formalin. The collected samples were fixed in 10% neutral buffered formalin, and dehydrated through graded alcohols and xilol before being embedded in paraffin wax. Several sections were cut from each tissue sample using a microtome Leica RM2035 (Leica Mycrosystems, Barcelona, Spain), mounted on treated microscope slides (Fisher Scientific Co) and subsequently stained with hematoxylin-eosin (HE) and Ziehl-Neelsen (ZN). The stained sections were examined under a microscope Olympus BX51 equipped with an Olympus U-CMAD3 digital camera for pathological lesions and for the presence of acid-fast bacteria (AFB). According to their location and extension, inflammatory cell type, and Mycobacterial load, PTB-associated histopathological lesions were classified as focal (focal and multifocal), and diffuse (paucibacillary, intermediate or multibacillary)^3^.

For immunohistochemistry, the Avidin-Biotin kit with peroxidase-based detection was used (Vector Laboratories, California, USA). Briefly, ICV sections were deparaffinised, hydrated and rinsed with tap water. Afterwards, slides were treated to quench the endogenous peroxidase by incubation with methanol containing 3% H_2_O_2_ for 10 min at room temperature and washed with water for 10 min. The tissue sections were incubated overnight at 4 °C with an anti-*ITLN2* polyclonal antibody (Aviva Systems Biology, San Diego, California), washed with TBS and incubated with a goat biotinylated anti-rabbit IgG (1:200 dilution). The sections were then incubated with VECTASTAIN Elite ABC Reagent for 30 min at room temperature. Finally, the sections were incubated with 3,3-diaminobenzidine tetrahydrochloride (DAB, Sigma, St. Louis, MO, USA) for 5 min and washed with TBS. The stained slides were dehydrated, mounted and studied under light microscopy.

For bacteriological culture, a pool (2 gr) of ileocecal lymph nodes, distal jejunal lymph node, ICV, and distal jejunum were decontaminated with 38 mL of hexa-decyl pyridinium chloride at a final concentration of 0.75% (Sigma, St. Louis, MO) and homogenized in a stomacher blender. After 30 min of incubation at room temperature, 15 mL of the suspension was transferred to a new tube and incubated overnight for decontamination and sedimentation. Approximately, 200 µl of the suspension was taken from the layer near the sediment and inoculated into two slants of Herrolds egg yolk medium (HEYM; Becton Dickinson, Sparks, MD) and into two slants of Lowenstein-Jensen medium (LJ; Difco, Detroit, MI), both supplemented with Mycobactin J (Allied Monitor, Fayette, MO) as previously described^51^. Bacterial load in tissues was classified as low (<10 cfu; estimated average 2 cfu/tube), medium (between 10 to 50 cfu, estimated average 20 cfu/tube), or heavy (>50 cfu; estimated average 200 cfu/tube). At the time of slaughter, feces were taken from the rectum of each animal and processed within 48 h after arrival at the laboratory. The fecal samples (2 g each) were decontaminated, blended in a stomacher, and cultured in HEYM and LJ, as previously described for tissue culture.

### Enzyme-linked immunosorbent assay (ELISA) for the detection of MAP-specific antibodies

Blood was collected from the coccygeal vein of each animal into 4.5 ml serum clot activator Vacutainer® tubes (Vacuette, Kremsmunster, Austria). Serum was separated after clotting by centrifugation (2500 × g for 20 min) and stored at −20 °C until use. The serum samples were tested using the Mycobacterium Paratuberculosis Antibody test kit (IDEXX laboratories, Oofddorp, The Netherlands) according to the manufacturer’s instructions. The optical density (OD) in each well was measured at 450 nm by an ELISA plate reader (model 680, Sigma, St. Lois, MO). The measured ODs were normalized and the results were expressed as a percentage of the positive control OD according to the following formula: % relative OD sample/OD positive control = 100 × [(OD sample_+Ag_ − OD sample_−Ag_)/(OD mean positive control_+Ag_ − OD mean positive control_−Ag_).

### Fecal real-time polymerase chain reaction (PCR)

Isolation of genomic DNA from feces was performed using the MagMax Total Nucleic Acid Isolation kit according to the manufacturer’s instructions (ThermoFisher Scientific, Lissieu, France). For detection of MAP DNA, the LSI VetMax Triplex real-time PCR was used according to the manufacturer’s instructions (ThermoFisher Scientific, Lissieu, France). The kit enables real-time PCR detection of *Map* IS900 and F57 genes in DNA extracted from feces, liquid cultures, and tissues or colonies. Real-time PCR amplifications were performed using the MX3000P Real-Time PCR system (Stratagene, San Diego, USA) detection system with the following conditions: 1 cycle at 50 °C for 2 min, 1 cycle of 95 °C for 10 min, 45 cycles of denaturation at 95 °C for 15 s, and annealing/extension at 60 °C for 60 s.

### Fecal quantitative real-time PCR (qPCR)

PCR-positive samples were also tested by real-time qPCR using the ParaTB Kuanti-VK kit following the manufacturer’s instructions (Vacunek, Bizkaia, Spain). The kit uses a F57 TagMan probe labeled with the fluorescent reporter dye 5-carboxyfluorescein (FAM) at the 5′end and primers that specifically amplify the single-copy F57 insertion sequence of MAP. Inhibition of the amplification reaction is ruled out by including in the master mix an internal plasmid control with specific primers and an internal hybridization probe labeled with 6-carboxy-4′,5′-dichloro-2′,7′-dimethosyfluorescein succinimidyl ester (JOE) at the 5′-end. This internal amplification control molecule is co-amplified alongside the F57 diagnostic target in a duplex format. Quantification of MAP titer (F57 copy numbers per gram of feces) was accomplished by preparing a standard curve using serial dilutions of a standard sample containing a known number of MAP DNA copies. Real-time qPCR amplifications were performed using the Step One Plus detection system (Applied Biosystems, Carlsbad, CA) with the following conditions: 1 cycle at 95 °C for 10 min, 45 cycles of denaturation at 95 °C for 15 s, and annealing/extensions at 60 °C for 60 s. The results were analyzed with the ABI Prism software version 1.4.

### RNA extraction and RNA-Seq library preparation

At the time of slaughter, PB samples were collected from the coccygeal vein of all the cows included in the study in PAXgene Blood RNA tubes (2.5 ml) (Qiagen, Hilden, Germany). Total RNA was extracted from the PB samples using the PAXgene blood RNA kit according to the manufacturer’s instructions (Qiagen, Hilden, Germany). For RNA isolation, 150–200 mg of ICV of all the animals were harvested and immediately submerged in 2 ml of RNA later (Sigma, St. Louis, MO). Purification of RNA was performed using the RNeasy Mini Kit according to the manufacturer’s instructions (Qiagen, Hilden, Germany). Residual genomic DNA was removed using DNase digestion with RNase-free DNase I Amplification grade following the recommended protocol (Invitrogen, Spain). Concentration and quality of the total RNAs were measured using an Agilent Bioanalyzer 2100 (Agilent Technologies, Santa Clara, CA, US). All samples had an RNA integrity value of 7 or greater. Approximately 250 ng of RNA were used for each RNA-Seq library creation using the Illumina NEBNext® Ultra Directional RNA Library preparation kit following the manufacturer’s instructions (Illumina Inc, CA, US). All RNA-Seq libraries were quantified using a Qubit® Fluorometer and doubled stranded DNA high Sensitivity Assay Kit (Invitrogen, Spain). RNA-Seq libraries quality was assessed using an Agilent Bioanalyzer and Agilent high sensitivity DNA chip to confirm that the insert sizes were 200–250 bp for all the individual libraries.

### RNA sequencing and bioinformatic analysis of RNA-Seq data

RNA-Seq libraries were single-end sequenced in a 1 × 75 format using an Illumina NextSeq500 sequencer at the Genomic Unit of the Scientific Park of Madrid, Spain. The raw reads were filtered by their length (minimum size 60 bp long) and percentage of ambiguous base N less than 5% using Prinseq-lite^52^. Trimmed reads were subsequently mapped to the *Bos Taurus* reference genome (Bos_taurus.UMD3.1. version 87) with TopHat mapper^53^. Reads were assigned to a gene if they were not multi-hit reads. The resulting alignment files were provided to Cufflinks to generate a transcriptome assembly for each condition. These assemblies were then merged together using Cuffmerge, which is included in the Cufflinks package. This merged assembly provided a uniform basis for calculating gene and transcript expression levels in each condition. The reads and merged assembly were fed to Cuffdiff which calculates expression levels and tested the statistical significance of each observed change in expression^54^. The fold change (log_2_ scale), P-values and false discovery rates (FDR) for each gene were obtained. The genes with a FDR-adjusted threshold <0.05 were considered differentially expressed (DE) when compared to the control group. To visualize and integrate all the data produced by the Cufflink analysis, CummeRbund was used for cluster analysis and for handling the transformation of Cuffdiff data into R statistical computing environment. RNA-Seq data have been deposited in the NCBI Gene Expression Omnibus (GEO) database under the accession number (GSE137395).

### Gene ontology (GO) and metabolic analysis of the DE genes obtained using RNA-Seq

To identify GO and metabolic routes involved in MAP infection, genes with a significant DE in each comparison were evaluated using the GOseq R Bioconductor^55–57^. To begin the analysis, GOseq quantifies the length bias present in the DE genes by calculating a probability weighting function (PWF) which gives the probability that a gene will be DE based on its length alone. Next, GOseq analysis requires the use of the Wallenius non-central hypergeometric distribution to approximate the null distribution for GO category membership and to calculate representation of GO categories amongst the set of DE genes. Using the Wallenius approximation, P-values for representation of the DE genes in each GO were generated and a P-value threshold <0.05 selected. GO analysis provided categories of genes involved in different biological processes (BP), molecular functions (MF) and those integral for different cell compartments (CC). KEGG pathways enrichment analysis was also performed using the STRING v11.0^58^.

### Protein-protein association networks

The DE genes with log_2_ fold changes >2 or < 2 were analyzed using the STRING database v10.5^59^. The STRING database aims to collect, score and integrate all known and predicted protein-protein association data. The basic interaction unit in the STRING database is the functional association, i.e. a link between two proteins that both contribute jointly to a specific function. Network nodes represent proteins and edges protein-protein interactions. For each interaction a combined and final score is computed based on seven evidence channels including neighborhood in the genome, gene fusions, co-occurrence across genomes, co-expression, experimental/biochemical data, association in curated databases, and co-mentioned in PubMed abstracts. Only functional interactions with a high confidence score (>0.7) were included in the networks.

## Supplementary information

Supplementary information.

## Data Availability

The datasets generated during the current study are available from the corresponding author on reasonable request.
