# Supplementary material for: RNA-Seq analysis of ileocecal valve and peripheral blood from Holstein cattle infected with Mycobacterium avium subsp. paratuberculosis revealed dysregulation of the CXCL8/IL8 signaling pathway
Source: Sci Rep. 2019 Oct 16;9:14845. doi: 10.1038/s41598-019-51328-0 (PMC6795908; doi:10.1038/s41598-019-51328-0)

SUPPLEMENTARY INFORMATION

RNA-Seq analysis of ileocecal valve and peripheral blood from Holstein cattle infected with *Mycobacterium avium* subsp. *paratuberculosis* revealed dysregulation of the CXCL8/IL8 signaling pathway

Marta Alonso-Hearn [1*], Maria Canive [1], Cristina Blanco-Vazquez [2], Rosana Torremocha[3], Ana Balseiro [2], Javier Amado [4], Endika Varela-Martinez [5], Ricardo Ramos [3], Begoña M. Jugo [5] and Rosa Casais [2]

1 NEIKER-Instituto Vasco de Investigación y Desarrollo Agrario, Animal Health Department, Derio, Bizkaia, Spain,
2 SERIDA, Servicio Regional de Investigación y Desarrollo Agroalimentario, Centro de Biotecnología Animal, Deva, Asturias, Spain,
3 Scientific Park of Madrid, Genomic Unit, Campus de Cantoblanco, Madrid, Spain,
4 LSAPA, Animal Health Laboratory of the Principality of Asturias, Department of Microbiology, Gijón, Asturias, Spain
5 University of the Basque Country (UPV-EHU), Department of Genetics, Physical Anthropology and Animal Physiology, Faculty of Science and Technology, Bilbao

Supplementary Table 1. Biological samples and experimental replicates RNA Seq data.

Cow ID	Replicate	Sample	Total number of reads	Reds after trimming	Reads after mapping	Mapped reads (%)	Multiple alignments	Multiple alignments (%)
1	_	PB	25378997	25321021	23442982	92,6	1013576	4.0
1	_	ICV	22485395	22448313	20778436	92.6	960728	4.3
2	1	PB	27430862	26221879	24731206	94.3	1348272	5.5
2	2	PB	25423706	24274388	22928902	94.5	1175411	5.1
2	1	ICV	21955291	20865686	19323967	92.6	789040	4.1
2	2	ICV	20328572	19424275	18014936	92.7	883206	4.9
3	_	PB	26328834	26269342	24364551	92.8	1323677	5.0
3	_	ICV	22553960	22509141	20747321	92.2	1048927	4.7
4	_	PB	22298387	22246905	20498481	92.1	873110	3.9
4	_	ICV	21880358	21833012	20326901	93.1	965338	5.2
5	_	PB	26322126	26257852	23915158	91.1	965338	5.7
5	_	ICV	23278256	23231904	21264647	91.5	1096951	4.7
6	1	PB	20051147	17459925	16706238	95.7	1033345	6.2
6	2	PB	20309821	17681238	16935538	95.8	1097540	6.5
6	1	ICV	18754089	17915042	16608514	92.7	815870	4.9
6	2	ICV	20982964	20028587	18542443	92.6	1196745	6.5
7	_	PB	24093748	24039957	22311230	92.8	830200	3.5
7	_	ICV	20544146	20515344	18971171	92.5	927826	4.5
8	1	PB	20329228	17660831	16867545	95.5	770714	4.6
8	2	PB	21073654	18326128	17481457	95.4	762102	4.4
8	1	ICV	20096314	19197461	18004883	93.8	751745	4.2
8	2	ICV	23087897	21995135	20627314	93.8	829144	4.0
9	_	PB	15868370	15831194	14637126	92.5	810996	5.1
9	_	ICV	21946355	21902385	20028060	91.4	1008152	4.6
10	_	PB	23181092	22173624	20866802	94.1	1217332	5.8
10	_	ICV	17870903	17147527	15779357	92.0	816491	5.2
11	1	PB	29153476	27883749	26522168	95.1	1227177	4.6
11	2	PB	24411524	23359067	22248361	95.2	955078	4.3
11	1	ICV	17831469	17102361	15725642	92.0	806102	5.1
11	2	ICV	26194068	25092879	23191445	92.4	1253006	5.4
12	1	PB	19426072	16928040	16147240	95.4	1101792	6.8
12	2	PB	21180755	18462166	17604588	95.4	1177815	6.7
12	1	ICV	26198482	25071815	23346127	93.1	1286444	5.5
12	2	ICV	24072525	23043123	21436647	93.0	1245385	5.8
13	_	PB	19591604	19543700	17648053	90.3	993927	5.1
13	_	ICV	18725483	18661911	16790989	90.0	965338	5.2
14	_	PB	22205068	22154209	20330845	91.8	965338	5.2
14	_	ICV	25017698	24974498	22905236	91.7	1239203	5.0
Mean			22312176	21448832	19963224	93.1	1013904.8	5.0

PB: Peripheral blood; ICV: Ileocecal valve

Supplementary figure 1. Volcano plots showing the DE genes (log_2 fold change versus –log (p value) for each sample and correspondent replica. The absence of red spots in the plots indicated the lack of DE genes between each sample and corresponding replica.

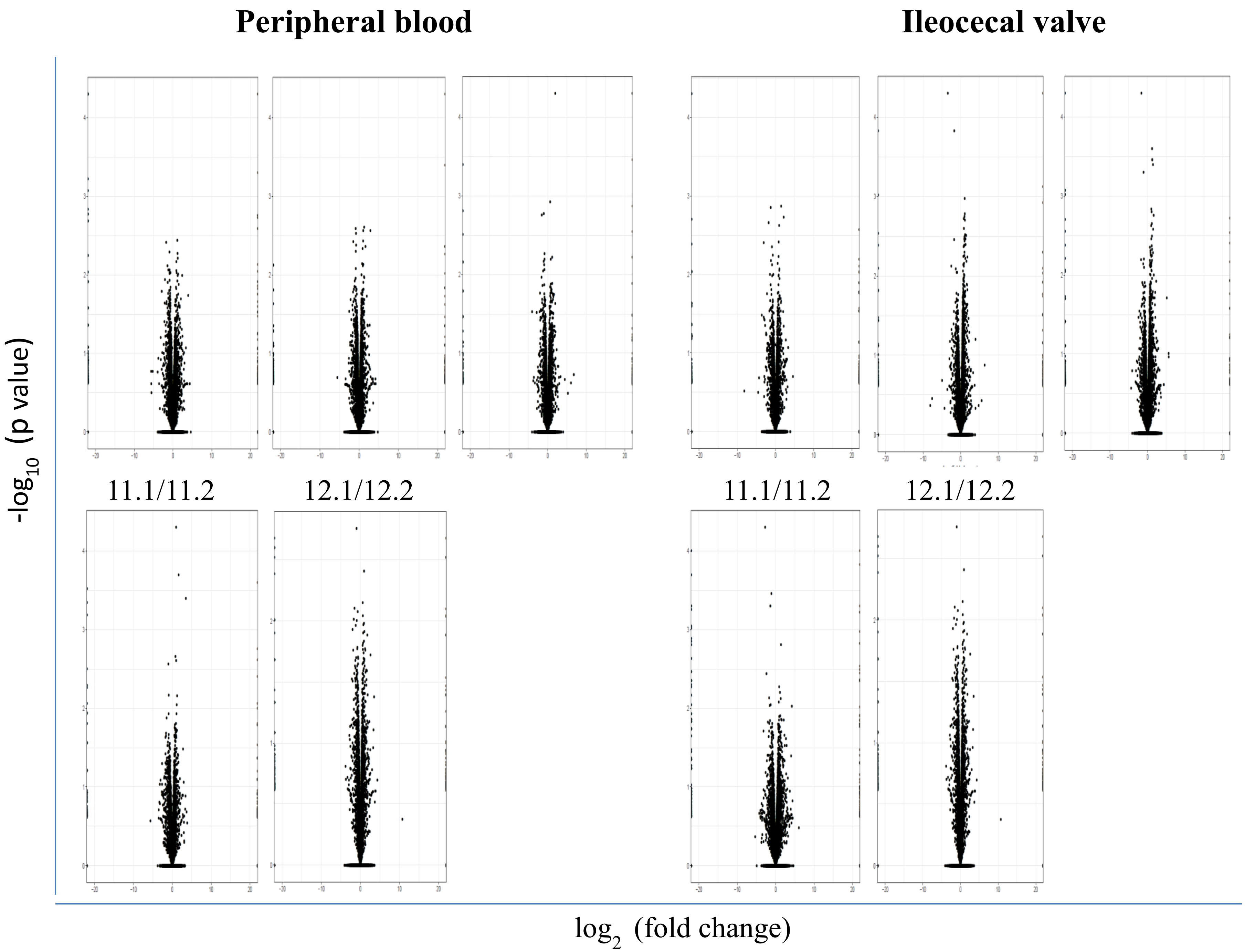

Supplement: Supplementary file 1 — Supplementary information. [file 41598_2019_51328_MOESM1_ESM.pdf]
